# The Quality of Mother–Child Feeding Interactions Predicts Psychopathological Symptoms in Offspring and Mothers Seven Years Later: A Longitudinal Study on the General Population

**DOI:** 10.3390/jcm12247668

**Published:** 2023-12-13

**Authors:** Silvia Cimino, Federica Andrei, Leonardo De Pascalis, Elena Trombini, Renata Tambelli, Luca Cerniglia

**Affiliations:** 1 Department of Dynamic and Clinical Psychology, Sapienza University of Rome, 00161 Rome, Italy; silvia.cimino@uniroma1.it (S.C.); renata.tambelli@uniroma1.it (R.T.); 2 Department of Psychology “Renzo Canestrari”, Alma Mater Studiorum, University of Bologna, 40126 Bologna, Italy; federica.andrei2@unibo.it (F.A.); leonardo.depascalis@unibo.it (L.D.P.); elena.trombini@unibo.it (E.T.); 3 Faculty of Psychology, International Telematic University Uninettuno, 00186 Rome, Italy

**Keywords:** feeding interactions, maternal psychopathology, internalizing symptoms, externalizing symptoms, developmental psychopathology

## Abstract

The increased risk of internalizing and externalizing symptoms in children has been observed in the presence of maternal psychopathology. This study aimed to investigate a potential pathway involving the quality of early interactions between mothers and their children. A sample of 150 mother–child dyads underwent assessment when the children were 3 years old and around the age of 10. Video recordings of feeding exchanges between mothers and children were analyzed to evaluate the quality of mother–child interactions. Maternal psychopathology and child internalizing and externalizing symptoms were measured through self-report and report-form measures completed by mothers. The quality of mother–child feeding interactions at three years of age significantly differentiated (*p* < 0.001), eight years later, between mothers at high and low psychopathological risk and between children exhibiting clinical and subclinical internalizing symptoms. Clinically relevant child symptoms were notably more prevalent when the mother–child interaction quality at three years of age was maladaptive, particularly in the context of concurrent high maternal psychopathological risk. The study findings underscore the importance of focusing on the early quality of mother–child feeding interactions to identify potential situations of maternal and child clinical risk for the development of psychopathological symptoms and to guide preemptive measures and policies.

## 1. Introduction

According to the developmental psychopathology perspective [1], the onset of emotional-behavioral problems in children can occur early in life, and a large number of studies have thus focused on preschool psychological difficulties, with the goal of identifying potentially problematic developmental pathways and organizing possible interventions [2]. Nevertheless, diagnosing and detecting symptoms in very young children can be particularly problematic, especially in normative samples, and distinguishing internalizing (e.g., withdrawal and anxiety) from externalizing syndromes (e.g., aggressiveness and hyperactivity) has emerged as a challenging task [3,4].

Despite these challenges, several authors have explored the epidemiology of maternal psychopathology in the general population, discovering substantial correlations with maladaptive child outcomes and finding both homotypic (i.e., continuation of a specific clinical phenomenon through time without significant phenotypic change) and heterotypic (i.e., presentations of multiple clinical forms across time) continuity [5,6,7]. In these studies, maternal psychopathology has been shown to be significantly associated with children’s internalizing and externalizing symptoms through several underpinning mechanisms [8], such as maternal- and child-shared genetic predispositions to psychopathology, disruptions in parenting that may contribute to offspring symptoms, child exposure to maternal distorted cognitions and/or stressful environments, and a lack of social support for the family as a whole [9,10].

This field of study has used both maternal clinical diagnoses and continuous measures of psychopathological risk focusing on specific problems (e.g., maternal depression or anxiety) or evaluations of the potential negative impact of parental difficulties of greater complexity (e.g., general psychological distress and outcomes of traumatic experiences) [11]. Moreover, some authors [12,13] have proposed that the maladaptive influence of specific diagnoses, such as maternal depression, can predict emotional-behavioral problems in children even when other relevant variables (e.g., other parents’ characteristics, marital discord, socioeconomic status) are controlled for. It has also been demonstrated that when a broader range of maternal psychopathology is considered in such studies, more useful information for prevention and early intervention programs can be captured [14], and children of mothers with co-occurring psychopathologies experience more severe symptoms and poorer psychosocial functioning [15].

While maternal psychopathology has been linked to child externalizing and internalizing problems, the bulk of research examining the transmission of psychopathological risk from mothers to children has used clinical samples comprising women with explicit diagnoses, leaving normative and sub-threshold populations relatively understudied [8,16,17]. Furthermore, the vast majority of studies have been carried out on clinical samples that typically show severe symptoms and frequently provide non-generalizable results. An interesting study on a normative sample found that infant exposure to maternal psychopathology for at least a year, even when maternal symptoms were moderate, predicted the probability of psychopathology in their offspring [18].

This field of study has also been enriched by research on parent-child interactions [19,20,21,22]. The quality of child social interactions with their primary caretakers during activities such as feeding and play in the first years of life is crucial for the adaptive or maladaptive nature of outcomes in later years [22,23]. It has been posited that children may not be able to learn how to regulate their emotions and may eventually exhibit maladaptive emotional and behavioral symptoms if the dyad is unable to mutually attune and share sensitive interactions, especially during feeding [24,25,26,27]. Previous literature has shown that specific problematic symptoms in maternal functioning can be related to low-quality feeding interactions with their children [28]. For instance, depression and anxiety symptoms in mothers have been found to be associated with maladaptive patterns of parent–child interactions. Obsessive-compulsive maternal symptoms, on the other hand, have been demonstrated to be linked to distorted maternal perceptions, repetitive negative thoughts, and obsessions about their child’s eating, which can have a detrimental impact on the quality of parent-child feeding exchanges.

### The Present Study

Notably, maternal symptoms can be considered both outcomes of a low-quality relationship and predictors of poor interactions. Therefore, there is a need for longitudinal studies to clarify the causal effects between variables. Therefore, this study aimed to assess the possible associations between maternal psychopathological risk and offspring maladaptive symptoms, considering the quality of feeding interactions.

Three hypotheses were tested: firstly, that the mother–infant interaction quality at three years of child age would distinguish low- and high-risk mothers, controlling for maternal age; secondly, that interaction quality at three years of age would distinguish low- and high-risk children, controlling for maternal age, child gender, and maternal level of psychopathology; and lastly, that a higher number of both high-risk mothers and children with clinically relevant symptoms would be detected at ten years of child age, when a maladaptive mother–child interaction quality was observed at three years of age.

## 2. Materials and Methods

### 2.1. Procedure

In 2012, we conducted a project in line with the developmental psychopathology theoretical framework, with the aim of informing families in the general population about the importance of the quality of feeding interactions for the psychological development of offspring at three years of age. Around the age of three, children are in a critical period of development characterized by rapid cognitive, emotional, and social growth, and they learn to more efficiently regulate their emotions in their interactions with peers and caregivers. Assessing mother–child interactions during this time allows for an understanding of how these interactions contribute to the child’s later emotional/behavioral functioning.

This project used video-recorded feeding interactions to determine the adaptive or maladaptive quality of exchanges between mothers and children. In 2020, and over the course of one year, the same team organized a new campaign to sensitize the general population in Central Italy about the possible associations between maternal psychopathological risk and offspring maladaptive symptoms, and a number of dyads included in the sample recruited in 2012 were reached by the campaign conducted in 2020, due to the shared territorial reach of the two projects. Around the age of 10, children are on the cusp of adolescence. Assessing emotional and behavioral functioning during this transition period provides insights into how well they would navigate the challenges associated with adolescence, such as changes in identity, social dynamics, and increased academic demands.

#### 2.1.1. Sample

The sample of the present study, thus, comprised 150 mother–child dyads (female offspring *n* = 84; mean age = 10.31, SD = 1.01; mothers’ mean age = 39.41, SD = 2.28). At the time of the present project, the children were approximately 10 years of age, and, for the purposes of the current study, we focused on the possible associations between maternal psychopathological risk and offspring maladaptive symptoms, considering the quality of feeding interactions evaluated in 2012.

#### 2.1.2. Study Design

A longitudinal study was conducted to observe the variables described below. The guidelines of the Declaration of Helsinki and the recommendations of good clinical practice (Document 111/3976/88 of July 1990) were taken into account in the conduct of the research. The ethics committee of Sapienza University (authorization n. 0811/2020-07/2019) approved the study protocol. The dyads were recruited over the course of one year in the general population with a consecutive sampling method. This was carried out following the aims of a prevention and intervention project focusing on families with preschool children [29]. A team of professional psychologists explained to mothers the study goals and methodology, how the data would be used, and obtained written informed consent.

The 150 dyads belonged to middle socioeconomic status (SES) households (93%). Ninety-six percent of mothers and children belonged to families with both parents present in the household; 92% of children attended kindergarten. All of the biological mothers and the children recruited for the current study were White Caucasian. A team of qualified psychologists visited the mothers and children at their homes to conduct the face-to-face assessment.

### 2.2. Measures

The Symptom Checklist-90 Revised [SCL-90/R] [30] was completed by the mothers to assess psychological risk. The SCL is a 90-item self-report symptom inventory measuring psychological symptoms and psychological distress rated on a Likert scale ranging from 0 (not at all) to 4 (extremely). The tool asks participants to report if they have experienced symptoms of somatization (e.g., headaches), obsessive-compulsivity (e.g., having to check and double-check what one has performed), interpersonal sensitivity (e.g., feeling that people are unfriendly), depression (e.g., feeling blue), anxiety (e.g., feeling fearful), hostility (e.g., having urges to beat, injure, or harm someone), phobic anxiety (e.g., fear of leaving one’s own house by oneself), paranoid ideation (e.g., persecutory beliefs concerning a perceived threat toward oneself), and psychoticism (e.g., having thoughts that are not one’s own) in the past week. The questionnaire also provides a global severity index (GSI). For the purposes of the present study, maternal symptoms were dichotomized into high vs. low risk according to the cutoff scores of the SCL (high-risk = above the cutoff point of 1 vs. low risk = below the cutoff point of 1). The SCL-90/R has shown good internal consistency in the present sample (Cronbach’s α = 0.83).

The Cognitive Behavioral Checklist (CBCL) is a questionnaire filled out by parents and caregivers to assess a child’s abilities and behavioral/emotional functioning over the last 2 months [31]. The CBCL/1.5-5 includes 100 items that provide scores for the Internalizing Problem Scale (the result of the sum of Emotionally Reactive, Anxious/Depressed, Somatic Complaints, and Withdrawn subscale scores) and for the Externalizing Problem Scale (consisting of Attention Problems and Aggressive Behavior subscales). For the purposes of the present study, the externalizing and internalizing symptoms dimensions of the CBCL were used and divided into subthreshold vs. clinically relevant symptoms. Cutoff scores for the CBCL are offered by the manual and are based on norms divided according to sex. This measure showed good psychometric properties in the present study, with a satisfactory internal consistency (alpha ranging from 0.65 to 0.96) and an excellent test–retest reliability (0.85).

The Observational Scale for Mother–Child Feeding Interactions (SVIA) is an Italian variant of the Feeding Scale [32,33] that may be used on children aged 1 to 36 months. It assesses interactive behaviors and identifies typical and/or dangerous relational types in parent-child feeding interactions. Parent–infant interactions during feeding are recorded for at least 20 min to use this measure, and a wide range of interactive mother–infant behaviors is then coded and analyzed. The SVIA consists of 41 items divided into four subscales: Parent’s affective state, interactive conflict, food refusal behavior, and dyad’s affective state. Scores are calculated using a Likert scale ranging from 0 (nothing) to 4 (a lot). Inter-rater agreement for SVIA items was outstanding (Pearson r = 0.9). Furthermore, the instrument demonstrated strong dependability in terms of internal consistency, with a Cronbach’s = 0.91. The means of the four subscales were also utilized to calculate a total unidimensional score in the current investigation. It has been proposed that the total of the four scores is suitable when using the scale to distinguish adaptive and maladaptive parent–child relationships (rather than detecting feeding issues in children) [32]. Higher ratings in this example characterized more problematic dyadic relationships during feeding. To differentiate clinical scores, Lucarelli and colleagues suggested a threshold of >54 (2 SDs from the mean in the Italian validation of the measure) [33].

### 2.3. Statistical Analysis

A preliminary screening of the data showed few data points missing for each instrument (3% for each instrument). Missing data were corrected using multiple imputations in SPSS software (Version 23.0). Bivariate associations were computed through Pearson’s *r* coefficient. Binary logistic regression analyses were used to assess whether the mother–child relationship quality, operationalized through the global score of the SVIA, predicted maternal and child psychopathological risk. Where maternal risk was the outcome variable, predictors in the model were maternal age and relationship quality. Where child symptoms were outcomes, child gender, maternal age, maternal psychopathological risk, and relationship quality were used as predictors.

Three-way loglinear analyses were also run to investigate the proportion of participants within the categories of maternal (2 levels obtained from the GSI’s scores; low- and high-risk) and child (2 levels obtained from the externalizing and internalizing symptoms dimensions of the CBCL (subthreshold vs. clinically relevant symptoms) psychopathological risk, according to relationship quality. To this end, the global score of the Feeding Scale was used to create two groups depending on whether the quality of mother–toddler interaction at three years of age was either adaptive (below the cutoff point = 54) or maladaptive (above the cutoff point = 54). Statistical analyses were performed using SPSS (version 26) for Windows (IBM, Armonk, NY, USA).

## 3. Results

Table 1 describes bivariate relationships among study variables. Positive correlations were detected between the global score of the Feeding Scale and the subscales of both the SCL and the CBCL, indicating more problematic interactions where mothers’ and children’s psychopathological symptoms are higher.

Table 2 displays the outcomes of logistic regression concerning maternal psychopathological risk. Through a chi-square goodness-of-fit test, it was found that the two predictors in the model effectively differentiated between mothers with high and low psychopathological risk across most dimensions of the SCL. The only exception pertained to paranoid ideation. Regarding relationship quality, the odds of having high psychopathological risk increased by a factor ranging from 1.01 to 1.10 for each one-unit rise in SVIA global scores, indicating a negative impact on relationship quality (higher scores denoting poorer quality).

Table 3 reveals logistic regression results for child symptoms. The chi-square goodness-of-fit tests demonstrated that the predictors in the model successfully distinguished between children with and without clinically relevant symptoms. However, it is noteworthy that the significance of relationship quality was observed solely in the case of internalizing symptoms. Here, the odds of having high internalizing symptoms increased by 1.04 for each one-unit rise in SVIA global scores, indicating a correlation with a lower-quality relationship.

Lastly, three-way loglinear analysis (Table 4) revealed a significant association among relationship quality, maternal psychopathological risk, and the clinical significance of child internalizing (*χ*^2^ = 14.42, *p* < 0.001) and externalizing symptoms (*χ*^2^ = 6.44, *p* = 0.011). Examination of the adjusted standardized residuals showed that dyads who presented maladaptive interaction patterns at three years of child age (*n* = 73) were more represented by mothers with higher psychopathological risk (*n* = 52; 71.2%) and children with clinically relevant internalizing (*n* = 32; 43.8%) and externalizing (*n* = 15; 20.5%) symptoms, compared to dyads who had adaptive interaction patterns (*n* = 76), which comprised lower numbers of high-risk mothers (*n* = 19; 25%), as well as of children with clinically relevant internalizing (*n* = 9; 11.8%) and externalizing (*n* = 5; 6.5%) symptoms.

## 4. Discussion

The present results showed that relationship quality assessed through feeding interactions at three years of child age discriminated between high- and low-risk mothers (Hypothesis 1) and between high- and low-risk children (Hypothesis 2) assessed eight years later, controlling for further pertinent characteristics. Furthermore, when mother–child relationship quality at three years was maladaptive, clinically relevant child symptoms were more prevalent, especially in combination with a higher maternal psychopathological risk (Hypothesis 3).

In particular, while these findings fully support Hypothesis 1, they provide only partial support for Hypothesis 2. In this study, relationship quality at 3 years significantly differentiated between subthreshold and clinically relevant child internalizing symptoms at 10 years of age, over and above maternal symptoms. This result confirms previous literature underscoring the enduring influence of the quality of early mother–child interactions on a child’s emotional well-being, transcending the influence of maternal symptoms alone. This highlights the critical role that the quality of the parent–child relationship plays in shaping the child’s psychological development in accordance with attachment theory, which posits that early parent–child interactions lay the foundation for a child’s emotional well-being [34]. For example, a child who feels emotionally connected and understood by their parent is more likely to develop effective coping mechanisms and resilience in the face of stressors. The results also emphasize the importance of addressing and understanding the nuances of parent–child interactions, particularly during formative years, as a key factor in predicting and preventing the emergence of internalizing symptoms in later childhood. Such findings contribute to the growing body of evidence emphasizing the significance of early relational experiences in shaping the mental health trajectory of children [35]. Moreover, this result goes beyond the traditional focus on maternal symptoms alone, emphasizing that the quality of parent–child interactions independently contributes to a child’s psychological development. For instance, even if a mother experiences symptoms of psychopathology, a nurturing and positive parent-child relationship can act as a protective factor, mitigating the impact of maternal symptoms on the child’s mental health. However, and differing from previous evidence [36], relationship quality did not play a significant role in the prediction of externalizing symptoms, notwithstanding their association. This unexpected finding challenges prevailing assumptions regarding the direct and lasting impact of early parent–child interactions on later externalizing behaviors [37,38,39]. It suggests a more intricate interplay of variables in the development of externalizing symptoms, wherein factors beyond the quality of early relationships may come into play. This result underscores the importance of considering a broader range of influences, such as peer relationships, school environments, or genetic/epigenetic predispositions, in understanding the emergence of externalizing behaviors in later childhood [40], which were not considered in this study. Further research could bring data to test this result; meanwhile, we can speculate that this result could be explained by taking into account the content of the tools that were used for the assessment of both child symptoms and the quality of mother–child feeding interactions. While child symptoms were operationalized through the CBCL, which does not provide a comprehensive representation of externalizing problems [41], relationship quality was operationalized through a tool that may easily predict symptoms pertaining to the domain of emotional, rather than behavioral, disturbance. At the same time, it can be hypothesized that, for example, if interaction dynamics at three years of age were characterized by intrusiveness, lack of reciprocity, and negative affectivity, in the course of time children may have shown avoidance, withdrawal, and emotional dysregulation, which may eventually increase the risk for the development of internalizing, rather than externalizing, problems [24,25,26,27]. According to our results, this may happen more frequently if mothers present high levels of psychological disturbance [42].

Overall, compared to previous research, which is mostly based upon clinical samples and self-report and retrospective measures of relationship quality, we used the SVIA which is an observational tool providing a comprehensive perspective on parent-child relationship quality as it includes not only parental (such as parental affective state) and child (such as their behavior during meal time) specific characteristics but also dyadic variables (such as conflict within the dyad and dyadic affective state). Some limitations should also be noted, particularly the assessment of both maternal and child symptoms through parental reports only, the lack of longitudinal measures on maternal and child symptoms, and the lack of information pertaining to relevant demographics (e.g., family composition, maternal occupational status, and level of education) as well as clinical factors. With this regard, variables pertaining to maternal psychological functioning at the time of the first assessment (e.g., early maternal relational traumas, depression levels) and data on fathers could offer further insights into the present study results [43]. In future research, the inclusion of multiple measures such as diagnostic interviews and observations, as well as the collection of pertinent risk/protective factors, would be important to obtain a more comprehensive picture of the longitudinal impact of relationship quality on maternal and child psychological well-being.

## 5. Conclusions

While these findings require replication on other samples and with further measures, they provide evidence to suggest that problems in mother–child relationships at three years of age may discriminate between maternal as well as child clinical risk years later. These results highlight the need to devote more attention to early interactions as useful indicators of the probability for both mothers and children to develop symptoms of psychological disturbance. This study also contributes valuable insights into the complex interplay between early mother–child relationship quality, maternal psychopathological risk, and subsequent child outcomes [44]. The findings support the hypothesis that relationship quality at three years discriminates between high- and low-risk mothers and children, with clinically relevant child symptoms more prevalent in cases of maladaptive mother–child interactions. While the study partially supports the hypothesis that relationship quality predicts internalizing symptoms at 10 years, it intriguingly reveals a non-significant role in predicting externalizing symptoms, challenging previous evidence [45,46,47]. Despite limitations, such as reliance on parental reports and a lack of longitudinal measures [48], the study underscores the importance of considering various measures, including diagnostic interviews and observations, to gain a more comprehensive understanding of the longitudinal impact of relationship quality on maternal and child psychological well-being. Future research should aim to incorporate additional variables, such as maternal psychological functioning and data on fathers, to further enrich our understanding of this intricate dynamic, although possible difficulties associated with a lack of funds and resources might hinder these studies. Online-based research could facilitate sample recruitment and measure administration, helping to reduce these problems.

## Figures and Tables

**Table 1:** Correlations among study variables for the entire sample.

Variable	1	2	3	4	5	6	7	8	9	10	11	12	13	14
Maternal age														
2. Feeding Scale global score	0.191 *													
3. Somatization	0.062	0.438 **												
4. Obsessive-compulsive	0.078	0.410 **	0.569 **											
5. Depression	0.153	0.336 **	−0.057	0.132										
6. Anxiety	−0.005	0.413 **	0.936 **	0.634 **	−0.105									
7. Hostility	0.197 *	0.385 **	−0.157	0.071	0.730 **	−0.226 **								
8. Phobic anxiety	0.065	0.358 **	0.921 **	0.590 **	−0.074	0.919 **	−0.231 **							
9. Psychoticism	0.124	0.293 **	−0.117	0.043	0.053	−0.186 *	0.620 **	−0.206 *						
10. Paranoid ideation	−0.099	0.163 *	0.134	0.259 **	0.431 **	0.126	0.426 **	0.104	0.185 *					
11. Interpersonal sensitivity	0.134	0.295 **	−0.089	0.139	0.762 **	−0.122	0.718 **	−0.140	0.268 **	0.456 **				
12. Global Severity Index	0.154	0.631 **	0.693 **	0.656 **	0.558 **	0.653 **	0.525 **	0.634 **	0.260 **	0.505 **	0.541 **			
13. Internalizing symptoms	0.148	0.661 **	0.525 **	0.435 **	0.437 **	0.438 **	0.490 **	0.445 **	0.354 **	0.309 **	0.399 **	0.781 **		
14. Externalizing symptoms	0.075	0.573 **	0.458 **	0.370 **	0.407 **	0.403 **	0.450 **	0.370 **	0.287 **	0.328 **	0.343 **	0.679 **	0.817 **	.

* *p* < 0.05, ** *p* < 0.001.

**Table 2:** Logistic regression analysis predicting maternal psychopathological risk with relationship quality and maternal age.

SCL Dimensions	B (SE)	OR	95% CI	Wald Statistic	*χ* ^2^	*p* Value
Global Severity Index					67.85	<0.001
Relationship quality	0.10 (0.02)	1.10	1.07–1.14	36.84		<0.001
Maternal age	0.02 (0.08)	1.01	0.86–1.20	0.04		0.833
Anxiety					28.57	<0.001
Relationship quality	0.06 (0.02)	1.01	1.04–1.10	19.83		<0.001
Maternal age	−0.06 (0.08)	0.94	0.81–1.09	0.62		0.430
Obsessive-compulsive					17.49	<0.001
Relationship quality	0.05 (0.01)	1.05	1.02–1.08	12.87		<0.001
Maternal age	0.03 (0.08)	1.03	0.89–1.19	0.13		0.715
Somatization					29.85	<0.001
Relationship quality	0.06 (0.14)	1.02	1.04–1.09	21.21		<0.001
Maternal age	−0.05 (0.07)	0.95	0.92–1.10	0.52		0.472
Hostility					27.48	<0.001
Relationship quality	0.05 (0.08)	1.05	1.02–1.08	15.49		<0.001
Maternal age	0.15 (0.01)	1.16	0.99–1.04	3.76		0.052
Phobic anxiety					17.07	<0.001
Relationship quality	0.04 (0.01)	1.04	1.02–1.07	13.46		<0.001
Maternal age	0.02 (0.07)	1.02	0.88–1.17	0.06		0.804
Depression					11.87	0.003
Relationship quality	0.04 (0.01)	1.04	1.01–1.07	7.44		0.006
Maternal age	0.09 (0.08)	1.10	0.93–1.29	1.18		0.278
Paranoid ideation					5.36	0.068
Relationship quality	0.02 (0.01)	1.02	0.99–1.05	2.01		0.156
Maternal age	−0.21 (0.11)	0.81	0.65–1.00	3.64		0.056
Interpersonal sensitivity					17.99	<0.001
Relationship quality	0.05 (0.02)	1.05	1.02–1.08	9.88		0.002
Maternal age	0.13 (0.08)	1.14	0.96–1.34	2.38		0.123
Psychoticism					10.84	0.004
Relationship quality	0.04 (0.02)	1.04	1.01–1.08	7.11		0.008
Maternal age	0.07 (0.09)	1.08	0.91–1.28	0.72		0.397

Note. SCL: Symptoms Checklist; B: estimated coefficient; SE: standard error; CI: confidential interval; OR: odds ratio.

**Table 3:** Logistic regression analysis predicting child’s psychopathological risk with relationship quality, maternal age, and child’s gender.

CBCL Dimensions	B (SE)	OR	95% CI	Wald Statistic	*χ* ^2^	*p* Value
Internalizing symptoms					49.23	<0.001
Child’s gender	−0.82 (0.44)	0.44	0.18–1.04	3.50		0.061
Maternal age	−0.10 (0.08)	0.90	0.77–1.07	1.43		0.231
GSI	2.26 (0.66)	9.62	2.64–35.12	11.75		<0.001
Relationship quality	0.04 (0.02)	1.04	1.00–1.07	4.30		0.038
Externalizing symptoms					26.61	<0.001
Child’s gender	−0.98 (0.55)	0.37	0.13–1.10	3.21		0.073
Maternal age	−0.15 (0.10)	0.86	0.71–1.04	2.39		0.122
GSI	2.33 (0.91)	10.25	1.73–60.67	6.57		0.010
Relationship quality	−0.51 (3.22)	1.03	0.98–1.08	1.70		0.193

Note. CBCL: Cognitive Behavioral Checklist; GSI: General Stress Index; B: estimated coefficient; SE: standard error; CI: confidential interval; OR: odds ratio.

**Table 4:** Distribution (%) of children with subthreshold and clinically relevant symptoms among high- and low-risk mothers divided by relationship quality at three years.

	Internalizing Child’s Symptoms		Total	Externalizing Child’s Symptoms		Total
	Subthreshold	Clinically Relevant		Subthreshold	Clinically Relevant	
**Adaptive RQ**						
Low risk mothers	57 (75)	–(0)	57 (75)	57 (75)	–(0)	57 (75)
High risk mothers	10 (13.2)	9 (11.8)	19 (25)	14 (18.4)	5 (6.6)	19 (25)
Total	67 (88.2)	9 (11.8)	76 (100)	71	5 (5.6)	76 (100)
**Maladaptive RQ**						
Low risk mothers	13 (17.8)	8 (11)	21 (28.8)	18 (24.7)	3 (4.1)	21 (28.8)
High risk mothers	28 (38.3)	24 (32.9)	52 (71.2)	40 (54.8)	12 (16.4)	52 (71.2)
Total	41 (56.2)	32 (43.8)	73 (100)	58 (79.5)	15 (20.5)	73 (100)

Note. RQ: relationship quality.

## Data Availability

The data presented in this study are available on request from the corresponding author.
